# A strategy for evaluating potential antiviral resistance to small molecule drugs and application to SARS-CoV-2

**DOI:** 10.1038/s41598-023-27649-6

**Published:** 2023-01-10

**Authors:** Karen Sargsyan, Karine Mazmanian, Carmay Lim

**Affiliations:** Institute of Biomedical Sciences, Academia Sinica, Taipei, 115 Taiwan

**Keywords:** Structural biology, Phylogenetics, Molecular evolution, Computational biology and bioinformatics, Protein analysis, Protein sequence analyses, Drug discovery, Medicinal chemistry

## Abstract

Alterations in viral fitness cannot be inferred from only mutagenesis studies of an isolated viral protein. To-date, no systematic analysis has been performed to identify mutations that improve virus fitness and reduce drug efficacy. We present a generic strategy to evaluate which viral mutations might diminish drug efficacy and applied it to assess how SARS-CoV-2 evolution may affect the efficacy of current approved/candidate small-molecule antivirals for M^pro^, PL^pro^, and RdRp. For each drug target, we determined the drug-interacting virus residues from available structures and the selection pressure of the virus residues from the SARS-CoV-2 genomes. This enabled the identification of promising drug target regions and small-molecule antivirals that the virus can develop resistance. Our strategy of utilizing sequence and structural information from genomic sequence and protein structure databanks can rapidly assess the fitness of any emerging virus variants and can aid antiviral drug design for future pathogens.

## Introduction

More than two years have passed since severe acute respiratory syndrome coronavirus 2 (SARS-CoV-2) caused a pandemic that has claimed > 6 million lives and affected the livelihood of billions by disrupting economy, education, and social interactions. Since its discovery, a flood of publications and preprints has emerged attempting to (i) find the origin of this virus and its evolution, (ii) describe the virus life cycle and pathogenesis, and (iii) develop prophylactic vaccines or treatments. However, little effort has been made to elucidate whether future mutations of SARS-CoV-2 proteins would annul the efficacy of approved/candidate drugs. Because alterations in viral fitness cannot be inferred from mutagenesis studies of an isolated viral protein, some drug-escaping mutants found by laborious scanning of virus mutants may not exist if they decreased virus fitness. On the other hand, *daily* large-scale analysis of virus gene sequences was previously unavailable, but is now available for SARS-CoV-2. Here, we present a generic strategy to assess which viral mutations will diminish drug efficacy using evolutionary analysis of virus gene sequences of protein-coding regions combined with biochemical/structural data on viral protein-drug interactions. We illustrate this strategy by using it to predict the near-term likelihood of SARS-CoV-2 resistance to current small molecule antivirals.

While prophylactic COVID-19 vaccines have been very successful, delivering efficient drugs to treat COVID-19 has proven to be much more difficult. Efforts directed at treating COVID-19 have focused mainly in developing drugs to curb over-reacting immune response or antivirals encompassing small molecules, peptides, and monoclonal antibodies (mAbs)^1^. To-date, six anti-SARS-CoV-2 mAbs, namely, (i) casirivimab + imdevimab (REGEN-COV), (ii) bamlanivimab + etesevimab, (iii) sotrovimab, (iv) tocilizumab (Actemra™), (v) tixagevimab + cilgavimab (Evusheld™), and (vi) bebtelovimab, in chronological order have been granted emergency use authorization (EUA) by the U.S. Food and Drug Administration (FDA). Among the many small molecule antivirals for SARS-CoV-2 that have been published, remdesivir (Veklury™), a ribonucleotide inhibitor of SARS-CoV-2 RNA-dependent RNA polymerase (RdRp or nsp12)^2,3^, is the first one approved by the FDA followed by baricitinib (Olumiant™), a selective inhibitor of host proteins, JAK1 and JAK2^4^. In addition, the FDA has granted EUA for ritonavir-boosted nirmatrelvir (Paxlovid™) and (Lagevrio™). Like remdesivir, molnupiravir also targets SARS-CoV-2 RdRp, but unlike remdesivir that acts as a delayed chain terminator to stall viral RNA synthesis^5^, molnupiravir serves as a mutagen to increase the virus mutation rate, leading to dysfunctional virus copies^6^. Nirmatrelvir is a reversible covalent inhibitor of SARS-CoV-2 main protease (M^pro^) and is boosted by ritonavir, an HIV-1 protease inhibitor that allows nirmatrelvir to remain active longer by inhibiting its cytochrome P450 3A-mediated metabolism^7^.

In the course of evolution, a virus will undergo mutations to propagate the spread of beneficial alleles (positive/diversifying selection) or hinder the spread of deleterious alleles (negative/purifying selection)^8^. Hence, certain mutations of SARS-CoV-2 proteins might reduce drug efficacy, posing a major concern. Indeed, the numerous mutations in the spike protein of the current circulating Omicron variant have significantly reduced the efficacy of REGEN-COV, bamlanivimab + etesevimab, and sotrovimab, causing the cessation of these three mAb therapeutics in the United States, whereas bebtelovimab has shown reduced efficacy against the Mu variant. Attempts have been made to determine mutations in the SARS-CoV-2 spike *trimeric* glycoprotein that escape neutralizing antibodies by creating mutants, expressing them, and determining if they affect the native virus fold and function and if not, how they affect antibody binding^9–11^. The results depend on (i) the coverage of all possible amino acid (aa) mutations of a given viral protein, (ii) whether the expression system expresses the viral protein in its functional, native oligomeric/glycosylated state, and (iii) the sensitivity of the binding assays. Due to the need to produce numerous viral mutant proteins in an isolated lab facility, few such studies have been completed. Furthermore, mutation of a certain SARS-CoV-2 protein may affect its interactions with other viral proteins and affect SARS-CoV-2 fitness.

In addition, several in silico studies^12–18^ using tools such as sequence analysis, structure modeling of SARS-CoV-2 variants, and molecular dynamics/docking simulations have predicted mutations of a *specific* viral protein that may alter its structure/flexibility and thus susceptibility to certain drugs. However, to our knowledge, no systematic analysis has been performed to assess if SARS-CoV-2 mutations are under positive/negative selection, which would alter drug efficacy in different ways: If mutations of drug-interacting residues of a given viral protein are under negative selection, they would be expected to revert to prevent harming the virus; hence, such substitutions may not escape current inhibitors in the near term. On the contrary, if they are under positive selection, they would be of great concern, as they would improve viral fitness and may negate the drug action.

Here, we present a strategy to evaluate which viral mutations might diminish drug efficacy by determining the drug-interacting virus residues from 3D structures and classifying their selection pressure using evolutionary information from genome sequences. A residue is deemed to be under positive (or negative) selection if it mutates faster (or slower) than would be expected by neutral drift alone. We then apply our strategy to predict the likelihood of viral resistance to current approved/candidate small-molecule drugs for SARS-CoV-2 proteins available from the scientific literature. This is timely due to the availability of copious SARS-CoV-2 genome sequences and many 3D structures of SARS-CoV-2 protein/inhibitor complexes. Our results help to elucidate the current SARS-CoV-2 resistance potential towards approved/candidate small molecule drugs. As large genetic surveying capabilities have been established in most countries following the COVID-19 pandemic, our generic strategy can be used to help select antiviral candidates against other viruses for clinical development.

## Methods

### Selection of small molecule approved/candidates

To obtain small molecule SARS-CoV-2 inhibitors, we searched the PubMed database using the following keywords: “SARS-CoV-2, drug, target, or protein”. This yielded ~ 10,000 published papers and preprints as of September 2021. We reduced this number by excluding all papers with approved/candidate drugs targeting host proteins or biologics (e.g., polypeptides and mAbs) or drug candidates comprising a mixture of known and unknown compounds such as plant leaves and other traditional medicine elements. Furthermore, we excluded drug candidates with unknown viral protein targets or whose impact on the viral protein target or whole virus have not been experimentally verified such as those from in silico screening alone. However, we did not judge the quality of the experiments completed, but deemed direct virus inhibition and experimental assays showing that the inhibitor interacts as predicted with the viral protein target to be sufficient. Finally, we kept only those experimentally verified small molecule inhibitors whose interactions with viral protein residues are known from crystal/docked structures. Again, we did not judge the methods used to identify such drug-interacting residues such as the quality of the viral protein-inhibitor structure. Supplementary Table S1 lists the resulting drug candidates and their virus protein targets. We do not claim that this list is comprehensive, as a few drug candidates may be omitted due to the enormous number of publications; moreover, new drug candidates are continually being reported.

### Evolutionary analysis

Human host virus proteins and their coding sequences from both RefSeq and GenBank complete genomes^19^ were obtained from NCBI using the NCBI Datasets service (on January 11 2022). As the number of sequences grows rapidly each day, analysis became infeasible on full sequence datasets. Hence, for a given virus drug target protein, we randomly sampled 20,000 different virus protein sequences, which were aligned using MaffT v7.487^20^. Guided by the multiple protein sequence alignment, we then aligned the coding sequences of the virus drug target protein using the msa-codon tool from the HyPhy 2.5.32 (MP) package^21^. The resulting multiple nucleic sequence alignment was supplied to IQTree 2.1.3^22^ to build a phylogenetic tree for the virus protein target. The model used to estimate the tree is selected by IQTree during its optimization search. To analyze the selection pressure at each site of the virus protein target, we employed the Fixed Effects Likelihood (FEL) method in the HyPhy 2.5.32 (MP) package^21,23^, which estimates the nonsynonymous and synonymous substitution rate at each site. Default p-values (p < 0.1) were used as a threshold to classify selection as negative or positive. No analysis of recombination was performed as studies found moderate evidence of recombination events and some recombination events may be explained alternatively^24,25^. To confirm the stability of the results obtained by the above procedure, we performed a total of 10 rounds of sampling from the original database.

### Structural analysis

We extracted the drug-interacting viral residues from protein-drug structures with the best resolution in the Protein Data Bank (PDB)^26^, or, if such structures are absent, from published docked structures where the drug candidate has been docked to a known experimental structure of the protein. Due to the lack of experimental data on the absolute free energy contributions of individual residues to drug binding, we did not attempt to rank the importance of the drug-interacting viral residues. To present the evolutionary analysis results to researchers working on drug design in an accessible manner, we mapped our sequence-based data on negative and positive selection to crystallographic structures of the corresponding proteins using SIFTS^27^. PDB residue numbering was employed for the drug-binding residues.

## Results

By surveying the PubMed database, we identified 149 experimentally verified small-molecule inhibitors whose SARS-CoV-2 drug targets and drug-interacting viral residues are known. They include the FDA-approved drug, remdesivir, as well as EUA-approved nirmatrelvir but not molnupiravir since there is no molnupiravir-bound SARS-CoV-2 RdRp structure. Supplementary Table S1 lists for each viral protein target, the drug candidates, the PDB code of the viral protein/inhibitor complex and the drug-interacting SARS-CoV-2 residues.

Most of the drug candidates in Supplementary Table S1 target a specific viral protein. However, some of them can bind to multiple sites in the same protein. For example, YM155, an anti-cancer drug in clinical trials, is found in three disparate sites of papain-like protease (PL^pro^) in the crystal structure of SARS-CoV-2 PL^pro^–YM155 complex^28^. Six drug candidates; viz., suramin, quercetin, compounds **7** and **13**, ebselen and disulfiram, target more than one SARS-CoV-2 protein. Suramin, a highly negatively charged molecule that has been used to treat African sleeping sickness and river blindness, binds to both SARS-CoV-2 M^pro^ and RdRp. It is thought to act at an allosteric site in M^pro^, causing conformational changes that alter protease activity^29^. It can also bind to the RdRp active site, blocking the binding of both RNA template and primer strands^30^. Quercetin, identified as a SARS-CoV-2 M^pro^ competitive inhibitor by an activity-based experimental screening, binds to the M^pro^ catalytic site^31^ as well as the spike receptor-binding domain^32^. It exhibits a dose-dependent destabilizing effect on the protease stability and inhibits the interaction between spike and human angiotensin-converting enzyme 2^32^. Compounds **7** and **13**, found using pharmacophore-based virtual screening, are peptidomimetic inhibitors of M^pro^ and PL^pro^ as well as human furin protease^33^. Ebselen and disulfiram are Zn^2+^-ejecting compounds that can simultaneously target reactive cysteines (free or Zn^2+^-bound) in multiple SARS-CoV-2 nonstructural proteins (nsps) comprising a replication transcription complex that replicates and produces subgenomic mRNAs encoding accessory and structural proteins^34–36^. Notably, ebselen forms a covalent bond with the catalytic Cys in M^pro^, as seen in the 2.05 Å crystal structure of the ebselen bound to M^pro^^37^.

The results in Supplementary Table S1 show that efforts to develop SARS-CoV-2 antivirals have focused on (i) nsp5 M^pro^ (the most targeted protein), (ii) nsp3 PL^pro^ domain, and (iii) the nsp12 RdRp catalytic domain. Both M^pro^ and PL^pro^ are excised from the viral polyproteins (pp1a and pp1ab) by their own proteolytic activities. For each of these 3 drug target proteins, we outline below the viral protein functions, overall structure, and distinct binding sites/motifs from available structures in the Protein Data Bank (PDB)^26^. Then, we describe where the drug ligands bind and the selection pressure of the drug-binding residues, which are numbered according to the respective PDB structure rather than the coding sequence. We underscore those SARS-CoV-2 M^pro^, PL^pro^, and RdRp residues under positive selection, as they might affect drug efficacy based on their reported roles.

### SARS-CoV-2 M^pro^ (3CL^pro^ or nsp5)

The main protease (M^pro^), also called 3-chymotrypsin-like protease (3CL^pro^) or nsp5, is a cysteine protease that cleaves the two viral polyproteins into 16 constituent nsps that are crucial for viral replication and maturation. It is the most popular SARS-CoV-2 nsp drug target because (i) it plays a prerequisite role for viral replication, (ii) it has no human homolog but is conserved among coronaviruses, and (iii) it has unique cleavage specificity, cleaving sequences after a Gln, unlike known human cysteine proteases^38–41^. Thus, drugs targeting M^pro^ would have reduced off-target activities and thus less side effects^42^.

**Figure 1:**
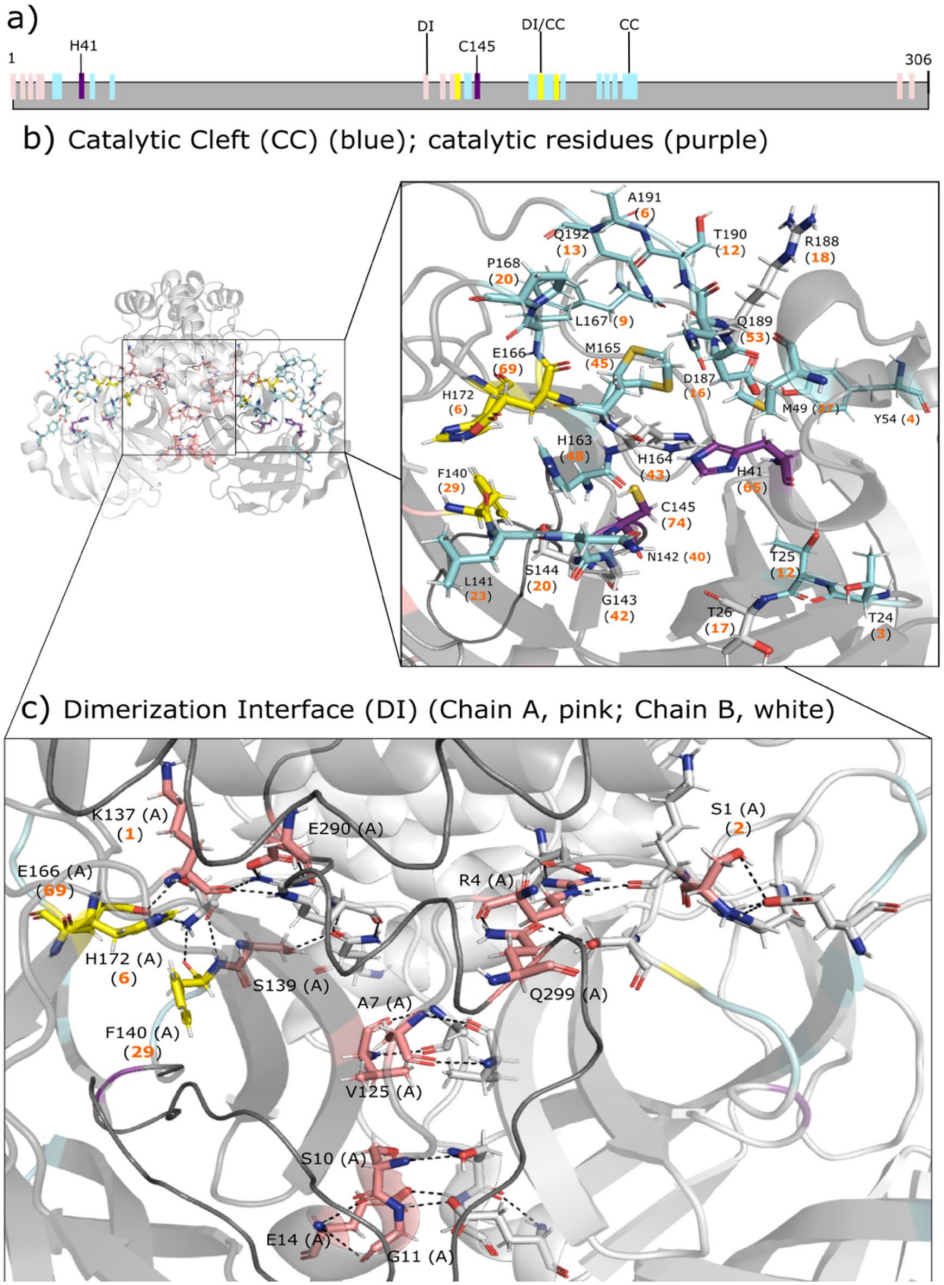
SARS-CoV-2 M^pro^ domain structure and binding sites. (**a**) Diagram showing the catalytic C145–H41 dyad (purple), the substrate-binding residues (light blue) in the catalytic cleft (CC), dimerization interface (DI) residues (pink), and residues shared by the catalytic cleft and dimer interface (yellow). (**b**,**c**) The 1.65-Å crystal structure of the M^pro^ homodimer (PDB 7ali) with one monomer in light gray and the other in gray. The inset shows the number of drugs (in parentheses) targeting a residue in the catalytic cleft (**b**) and at the dimer interface (**c**). Only residues from chain A are indicated for clarity.

Monomeric M^pro^ consists of an N-terminal finger (residues 1–7) and three domains: the chymotrypsin-like domain I (residues 8–101), the picornavirus 3C protease-like domain II (residues 102–184) and domain III (residues 201–306)^43^. Dimerization is needed for M^pro^ function, as interaction between the protomers, in particular the interaction between the N-terminal S1 of one protomer and E166 of the other promoter, keeps the enzyme in an active conformation^38^. Thus, the N-terminal finger, E166, and the unique catalytic C145–H41 dyad play a vital role in proteolytic activity. M^pro^ has *two* distinct binding regions (Fig. 1): (i) a substrate-binding site, containing the catalytic C145–H41 dyad, located in the cleft between domains I and II, and (ii) the dimerization interface involving residues from the N-terminal finger, the catalytic cleft and domain III^40,44–46^.

Figure 1b,c show the number of M^pro^ inhibitors in parentheses targeting (i) the catalytic C145–H41 dyad (purple), (ii) substrate-binding residues (light blue), (iii) dimerization interface residues (pink), and (iv) residues shared by the catalytic cleft and the dimer interface (yellow). All 94 inhibitors targeting M^pro^ including the EUA-approved drug nirmatrelvir (PF-07321332) bind in the catalytic cleft. They most frequently target the catalytic C145–H41 dyad (74 and 65 compounds) as well as E166 (69 compounds), which is important for dimerization. However, 3 of the 94 drug candidates (omeprazole, punicalagin, and chebulagic acid) also target two residues (S1 and K137) at the dimer interface. Punicalagin and chebulagic acid are also allosteric inhibitors of M^pro^ enzymatic activity^29,47^.

**Figure 2:**
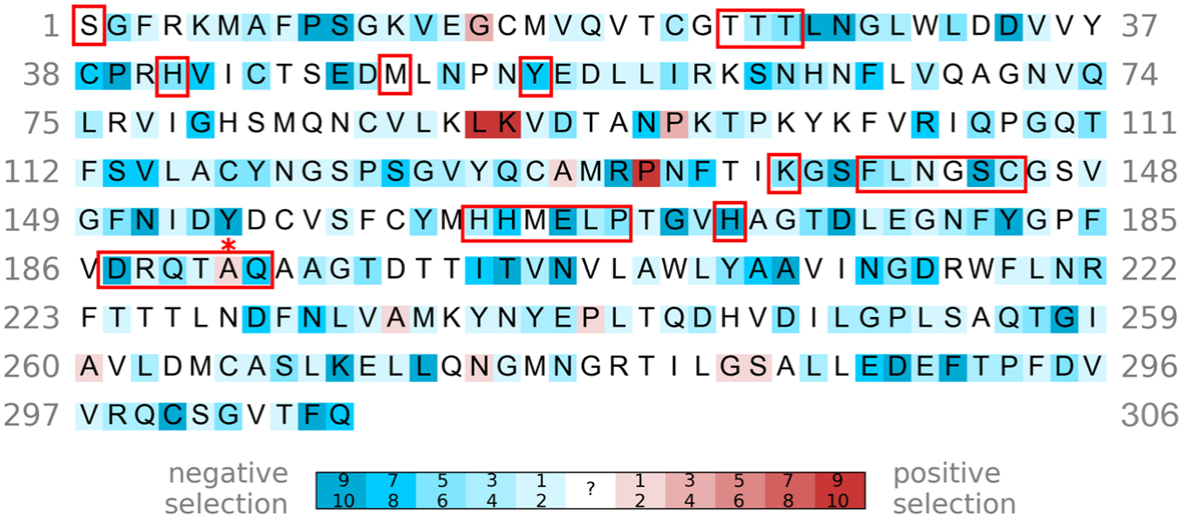
Selection pressure of SARS-CoV-2 M^pro^ residues. Out of 10 sampling rounds, the number of times a residue was found to exhibit evidence (p < 0.1) for negative and positive selection is indicated by increasing blue or red intensity, whereas a residue with no evidence to support negative or positive selection is in white. All drug-interacting residues are boxed and those under positive selection are indicated by asterisks.

Figure 2 depicts the SARS-CoV-2 M^pro^ residues that exhibit evidence (p < 0.1) for negative selection (blue) or positive selection (red) in any of the ten rounds of sampling or no evidence for negative/positive selection (white). For example, out of ten sampling rounds, the catalytic C145 showed evidence of negative selection in 4 rounds, but no evidence of positive/negative selection in the other rounds. Most of the residues targeted by the M^pro^ inhibitors^45,46^; viz., T25, T26, H41, Y54, K137, F140, L141, N142, S144, C145, H163, H164, E166, L167, P168, H172, D187, R188, Q189, T190, Q192, are under negative selection. The other drug-interacting residues (S1, T24, M49, G143, M165) show no evidence for negative/positive selection, but are highly conserved. Residues that are under positive selection do not directly interact with the M^pro^ inhibitors except for A191.

A191 displayed evidence of positive selection in 2 of the 10 sampling rounds. It is targeted by 6 drugs; viz., PF-00835231, efonidipine, nelfinavir, bisindolylmaleimide IX, as well as compounds **2a** and **151**. PF-00835231, a ketone-based covalent inhibitor, forms van der Waals interactions with the A191 backbone^48^. However, due to its low oral bioavailability, it has been superseded by the oral drug, PF-07321332 (EUA-approved nirmatrelvir), which does not interact with any residue under positive selection pressure. Interestingly, G15, K90, and P132, which are often mutated in current SARS-CoV-2 variants of concern^43^, are under positive selection. Since the mutation of K90 to Arg is expected to improve dimerization^43^, it may affect compounds that target the dimer interface.

### SARS-CoV-2 PL^pro^

**Figure 3:**
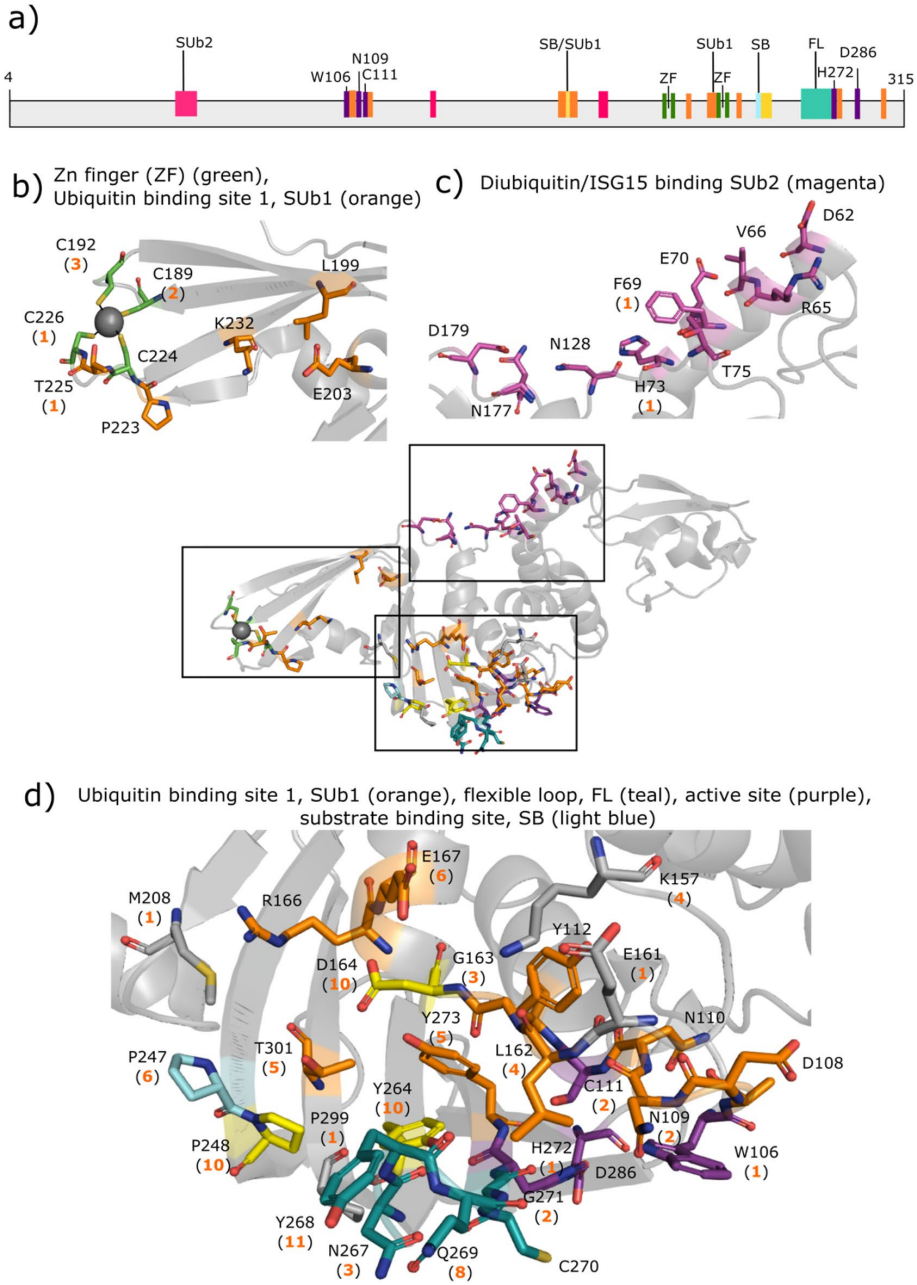
SARS-CoV-2 PL^pro^ domain structure and binding sites. (**a**) Diagram showing the active-site catalytic residues (W106, C111, H272, D286 in purple), the substrate-binding residues (SB, light blue), residues 267–271 in the flexible loop (FL, teal), residues in the SUb1 (orange) and SUb2 (magenta) subsites, and residues shared by the active-site cleft and the SUb1 subsite (yellow). The 1.90-Å crystal structure of apo PL^pro^ (PDB 7d7k)^28^ and the number of drugs (in parentheses) targeting residues in the Zn^2+^-binding site (**b**), the SUb2 subsite (**c**), and the active site, which overlaps with the SUb1 subsite (**d**).

SARS-CoV-2 nsp3-encoded PL^pro^ protease is also a popular drug target, as it is involved in viral replication and host immune response suppression and is conserved among coronaviruses^41,49^. This protease recognizes the LXGG↓(X) cleavage motif at the nsp1/2, nsp2/3, and nsp3/4 boundaries of the viral polyprotein and at the C-termini of host ubiquitin and interferon-stimulated gene 15 (ISG15)^50^. Hence, in addition to cleaving viral substrates, PL^pro^ also cleaves post-translational modifications on host proteins to evade antiviral immune responses^51^. Unlike M^pro^, PL^pro^ employs a catalytic *triad* (C111–H272–D286) and is catalytically active as a *monomer*. PL^pro^ consists of an N-terminal ubiquitin-like subdomain and a right-handed thumb-finger-palm catalytic unit^49^. It has *four* binding sites (Fig. 3a): a Zn^2+^-binding site, a viral substrate-binding channel, and two host ubiquitin/ISG15-binding subsites called SUb1 and SUb2^28,41,43,44,49,52^. The Zn^2+^-binding site, lined by 4 conserved cysteines (Fig. 3b), is essential for structural integrity and protease activity^53^. The SUb2 subsite consists of D62, R65–V66, F69–E70, H73, T75, N128, N177, and D179 (Fig. 3c). The SUb1 subsite consists of W106–Y112, E161–D164, R166–E167, L199, E203, P223, T225, K232, P248, Y264, Y268–G271, Y273, and T301 (Fig. 3d)^54^. Notably, W106 and N109 contribute to the stabilization of the oxyanion transition state of peptide hydrolysis^41^, whereas L162 and E167 are involved in interactions with host ISG15^55^. The SUb1 subsite partially overlaps with the viral substrate-binding channel containing the C111–H272–D286 catalytic triad, G163–D164, P247–P248, Y264, and a flexible loop termed BL2 (residues 267–271)^41,43,44,49,52^. The BL2 loop is important as it recognizes the LXGG motif in-between viral proteins and closes upon substrate/inhibitor binding^52^.

Most of the PL^pro^ inhibitors target the active-site cleft, 3 compounds target the Zn^2+^-binding site, and only one compound (YM155) is found in the SUb2-binding site (Fig. 3). Most of the drug candidates target residues involved in binding the substrate in the SUb1-binding site. In particular, Y268 is the most frequently drug-targeted residue (11 compounds), followed by D164, P248, and Y264 (10 compounds each), and Q269 (8 compounds). Two compounds, VIR250 and VIR251, are covalently bonded to the catalytic C111^56^.

**Figure 4:**
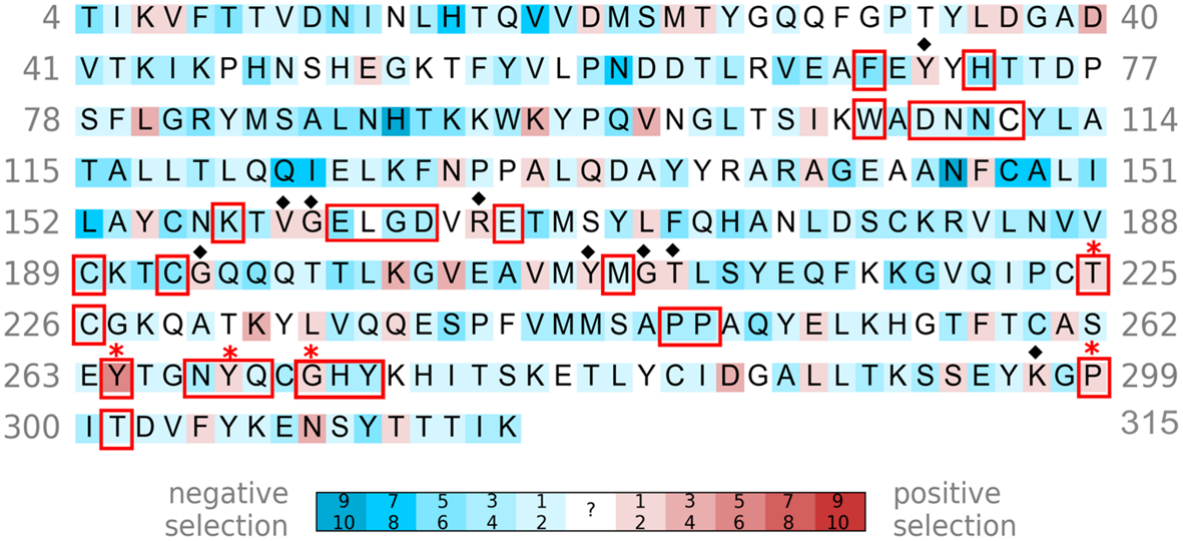
Selection pressure of SARS-CoV-2 PL^pro^ residues. PL^pro^ residues under increasing negative and positive selection are depicted by increasing blue and red intensity, respectively, whereas those with no evidence to support negative or positive selection are in white. All drug-interacting residues are boxed and those under positive selection are indicated by red asterisks. Residues under positive selection neighboring drug-interaction sites are indicated by black diamonds.

Comparison of Figs. 2 and 4 shows that there are more residues under positive selection (red residues) in PL^pro^ than there are in M^pro^. Nearly all the drug-interacting residues that are under positive selection are located in the SUb1 subsite, which binds host ubiquitin and ISG15 proteins. These residues include Y268, Y264, G271, and T225 which are targeted by 11,10, 2, and 1 inhibitor, respectively. Notably, Y268 in the BL2 loop can form hydrogen bonding and/or π-stacking interactions with the drug candidates; hence, its mutation could affect the BL2 loop conformation and attenuate drug interactions. Indeed, the mutation of SARS-CoV-2 PL^pro^ Y268 to Thr or Gly substantially reduced the inhibitory effect of the non-covalent inhibitor, GRL-0617^51^. Another drug-interacting residue under positive selection is P299, which forms hydrophobic contacts with only 1 drug candidate, XR8-24^57^. Interestingly, the 2.1 Å crystal structure of SARS-CoV-2 PL^pro^–YM155 complex (PDB 7D7L) shows YM155 forming van der Waals or hydrogen-bonding interactions with (i) C192, Q195, T225, and C226 in the Zn^2+^-binding site, (ii) P248, Y264, Y268, and Y273 in the viral substrate-binding channel, and (iii) F69 and H73 in the SUb2 subsite^28^. Although C192 and H73 are under negative selection, neighboring G193 and Y71, respectively, are under positive selection. Since G193, T225, Y264, Y268 and Y71 are under positive selection, their mutations may attenuate binding of YM155 to all 3 sites.

Apart from Y71 and G193, several other residues under positive selection are also near the drug-interacting residues. Positively charged K232 is near the negatively charged Zn^2+^-site (Fig. 3b), and its mutation to Gln present in the SARS-CoV-2 gamma variant of concern (K232Q) enhanced ubiquitin cleavage in vitro, which could affect the host immune response in infected cells^54^. R166 is near two popular drug-interacting acidic residues, D164 and E167, whereas (V159, G160), (Y207, G209, T210), and K297 are adjacent in sequence to E161, M208, and P299, respectively, which each interact with only one inhibitor (Fig. 3d). Surprisingly, D286 is under positive selection even though it is part of the catalytic triad. By forming a hydrogen bond with the H272 side chain, D286 serves to align H272 to act as a general acid/base during catalysis^43^. This role of D286 may be compensated by a buried water molecule as found in M^pro^, which lacks a third catalytic residue.

### RNA-dependent RNA polymerase (nsp-12)

The nsp12 RdRp is another key drug target because it is responsible for viral RNA synthesis, and is highly conserved among coronaviruses with no known mammalian homologs^16^. The nsp12 subunit consists of three domains: the N-terminal nidovirus RdRp-associated nucleotidyl-transferase domain (NiRAN, residues Q117–A250), the interface domain (residues L251–R365), and the finger–palm–thumb RdRp catalytic domain (residues L366–L932)^41^. By itself, nsp12 shows little or no polymerase activity, which requires the help of nsp7 and nsp8 cofactors to increase nsp12 binding to the template-primer RNA^5^. Two conserved Zn^2+^-binding motifs (H295, C301, C306, C310 and C487, H642, C645, C646) maintain the structural integrity of RdRp^5^. In addition to the two Zn^2+^-binding sites, seven conserved structural motifs (labelled *A*–*G*) in the RdRp catalytic domain are involved in binding the RNA template and primer strands and/or incoming nucleotide. During the template-directed RNA synthesis, the single-stranded RNA template passes along a groove clamped by motifs *F* (T538–V560) and *G* (K500–R513) and enters the active site composed of motifs *A–D*^58^. Motifs *A* (N611–M626) and *C* (F753–N767) contain the catalytic ^618^DX_4_D^623^ and ^759^SDD^761^ motifs, respectively, where the conserved acidic residues are involved in regulating catalytic activity and binding two catalytic Mg^2+^ ions^58^. Motif *B* (T680–T710) contains a flexible loop (S682–T686) involved in template binding and translocation of the nascent dsRNA^58^. Motif *E* (H810–K821) interacts with the primer RNA strand^5^, whereas motifs *D* (L775–E796) and *F* interact with the incoming NTP phosphate group^58^.

**Figure 5:**
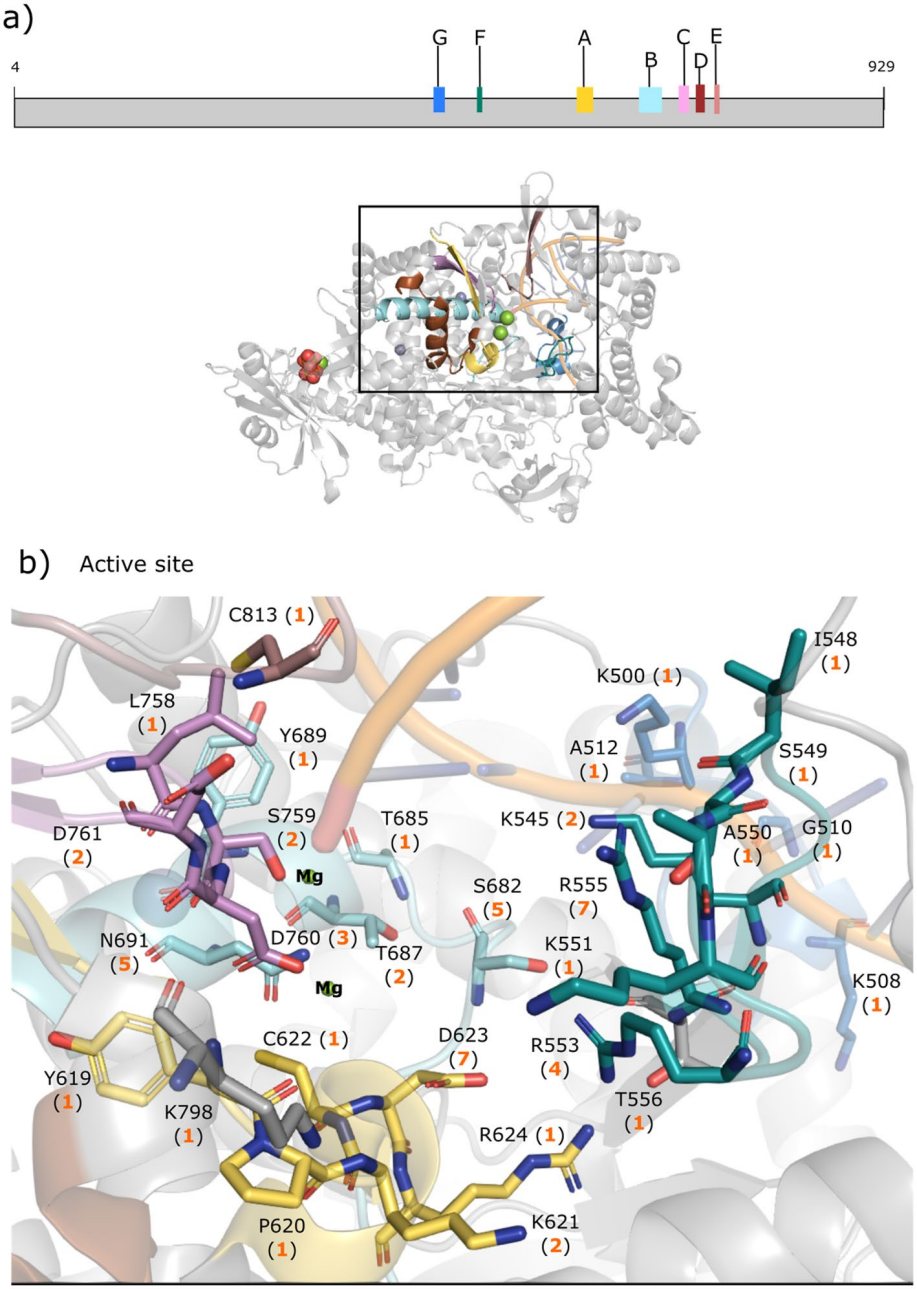
SARS-CoV-2 RdRp domain structure and binding sites. (**a**) Diagram showing the seven conserved motifs (A–G) in nsp12 and the cryo-EM structure (PDB 7aap). (**b**) The inset shows the number of drugs (in parentheses) targeting a residue belonging to one of the 7 conserved motifs (A–G)^58^.

Nearly all identified nsp12 drug candidates, including FDA-approved remdesivir, target residues comprising the conserved structural motifs in the nsp12 catalytic domain. They most frequently interact with positively charged R555 in motif F, which contacts the + 1 base of the primer strand RNA, negatively charged D623 in the catalytic ^618^DX_4_D^623^ motif as well as S682 and N691 in motif B (see Fig. 5). None of the nsp12 drug candidates identified bind to the two Zn^2+^-sites or motif D.

**Figure 6:**
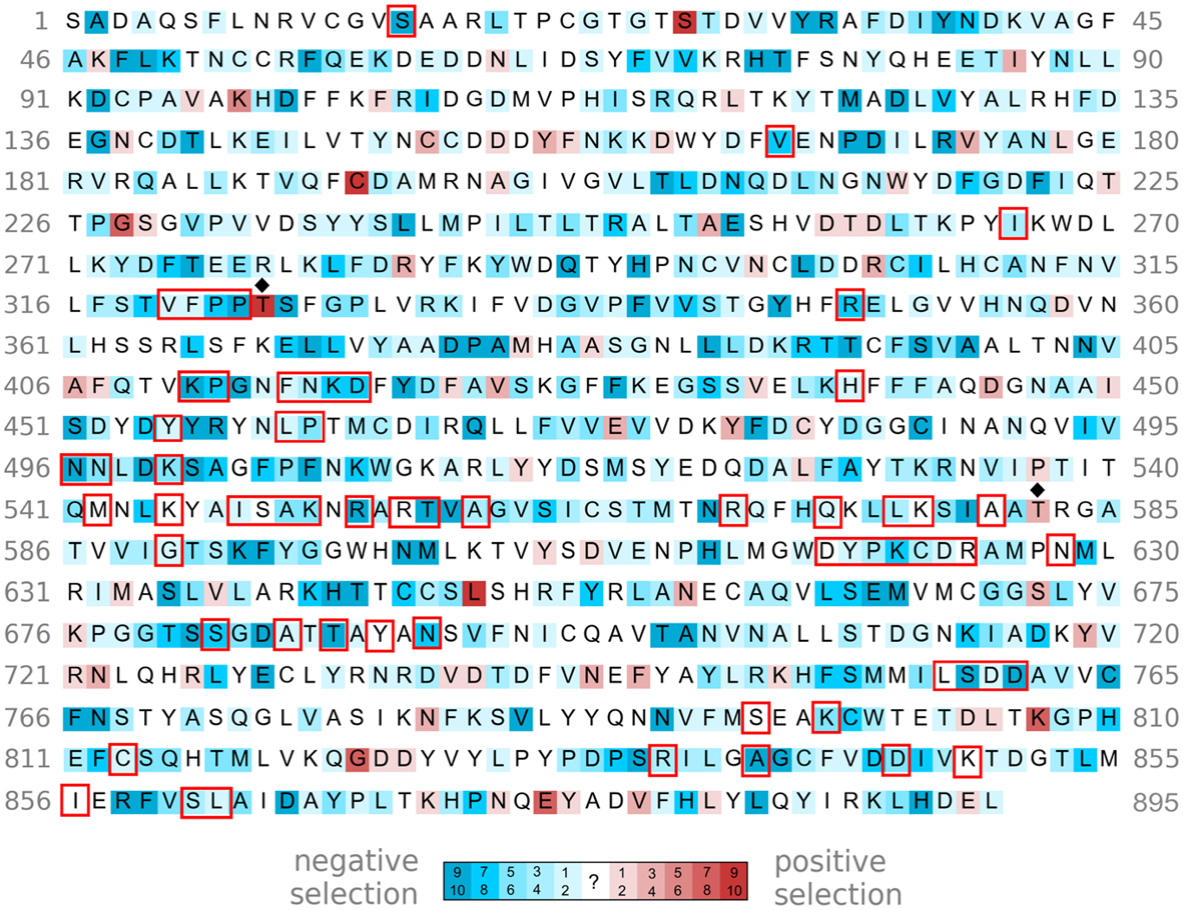
Selection pressure of SARS-CoV-2 RdRp residues. The sequence after L895 in the cryo-EM structure (PDB 7aap) has deletions or missing residues, and could not be reliably mapped to the aa residues from the gene sequences. RdRp residues under increasing negative and positive selection are depicted by increasing blue and red intensity, respectively, whereas those with no evidence to support negative or positive selection are in white. All drug-interacting residues are boxed and those neighboring residues under positive selection are indicated by black diamonds.

Most of the drug-interacting residues, in particular, the ^759^SDD^761^ catalytic residues are under negative selection (Fig. 6). Notably, S861, which plays a key role in the delayed chain termination mechanism of remdesivir, is under negative selection. However, R555, which is most frequently targeted by the SARS-CoV-2 RdRp inhibitors including remdesivir, show no evidence for either negative/positive selection. On the other hand, in vitro evolution studies have identified three nsp12 mutants, viz., S759A, V792I, and E802(A/D), to confer resistance to remdesivir^17,18,59^. However, S759 comprising the ^759^SDD^761^ catalytic motif and V792 are both under negative selection, suggesting that their mutations would decrease SARS-CoV-2 fitness. Although highly conserved E802 shows no evidence for either negative/positive selection, E802(A/D) mutants decreased viral replication relative to wild-type SARS-CoV-2 nsp12 in in vitro assays, indicating that E802 mutations impart a fitness cost^59^.

None of the drug-interacting SARS-CoV-2 RdRp residues are under positive selection; however, some are near residues that are under positive selection. For example, T324, which displayed evidence of positive selection all 10 sampling rounds, is next to two prolines (P322 and P323) that are predicted to interact with the inhibitor Taroxaz-104^60^. Another residue under positive selection, T582, is close to A580, which has packing interactions with suramin in the crystal structure of the SARS-CoV-2 RdRp bound to suramin (PDB 7d4f).

## Discussion

An important by-product of the COVID-19 pandemic is that most countries have established extended genome surveying capabilities to monitor and analyze changes in the viral genome. These surveying capabilities can be applied to future epidemics/pandemics. Therefore, we propose using information obtained from genomic databanks to support antiviral drug design. Herein, we illustrate how such data can be incorporated in the early stages of antiviral drug design by extracting evolutionary trends from a large-scale analysis of SARS-CoV-2 gene sequences. This enabled us to identify good SARS-CoV-2 drug target sites and drug candidates with a high probability of antiviral resistance in the *short* term. In contrast to our proposed strategy, previous studies generally employed conservation across the coronavirus family as a proxy to identify good viral drug target sites and associated high-frequency mutations with the likelihood of antiviral resistance; e.g., M^pro^ residues that are most prone to mutations have been assumed to be potential sites of resistance^46^. However, the mutation frequency seen in nonstructural proteins does not provide direct evidence for the likelihood of the mutation to be beneficial and not detrimental for the virus. We observed some variation at every residue position in our pool of viral sequences, so we can count mutations that do not improve viral fitness.

### Implications for SARS-CoV-2 drug targets and drug candidates

Among the three most popular SARS-CoV-2 drug targets, M^pro^ has the least number of residues showing positive selection, whereas PL^pro^ has the most (compare Figs. 2, 4 and 6). Therefore, targeting the M^pro^ or RdRp active site has more evolutionary support than targeting the PL^pro^ active site. Our results further suggest promising drug target regions comprising residues under negative selection that are not spatially near residues under positive selection. For example, the results for RdRp in Fig. 6 indicate two contiguous regions containing residues under negative selection (^494^IVNNLDKS^501^ and ^840^AGCFVDDIV^848^) and the closest residue under positive selection is > 8 Å.

Although residues under positive selection may not directly interact with a given drug, their mutations may regulate drug interactions allosterically and may confer drug resistance. Hence, drugs targeting residues/regions exhibiting negative selection in *multiple* essential viral proteins can better counter the dangers posed by new mutations than drugs targeting a single viral protein. Indeed, Zn^2+^-ejector drugs (ebselen, disulfiram) have been shown to simultaneously target the catalytic and/or Zn^2+^-bound cysteines in five SARS-CoV-2 proteins; viz., M^pro^^61^, PL^pro34^, nsp10 (a cofactor of nsp14 and nsp16)^34^, nsp13 RNA helicase/5′-phosphatase^35^, and nsp14 exonuclease domain^35^. In contrast to ebselen/disulfiram, peptidomimetic drug candidates cannot act on both M^pro^ and PL^pro^ simultaneously, as these two viral proteases have quite different substrate specificity^50^; hence their separate inhibitors have to be combined. To minimize the risk of resistance emergence and maximize potency, we propose combining multi-targeting clinically safe ebselen/disulfiram with potent inhibitors targeting M^pro^ and/or RdRp residues that are under negative selection. Indeed, the combination of ebselen/disulfiram targeting nsp3 PL^pro^, nsp5 M^pro^, nsp10, nsp13, and nsp14 with remdesivir targeting nsp12 RdRp has been shown to *synergistically* inhibit SARS-CoV-2 replication in Vero E6 cells^35^.

### Limitations

Owing to the lack of experimental data on the free energy contributions of individual viral residues to drug binding, we could not evaluate the impact of drug-interacting residues or their aa changes on drug binding. Note that the aa changes at a positive selection site do not impact drug binding equally, as some changes may totally abrogate the drug’s action, whereas others may only have a marginal impact on drug binding. Furthermore, it is the collective effect of all aa changes in a drug target protein that determines drug resistance. Note that the results in Figs. 2, 4, 6 are based on current SARS-CoV-2 gene sequences (till January 2022). Although mutations at sites under negative selection occur and may lead to drug-resistant viruses^62^, these naturally occurring variants under the viral fitness landscape described by the current data would likely be less fit. However, when more antivirals become approved and widely used, SARS-CoV-2 may acquire mutations to become resistant to antiviral therapy. Despite the lack of nirmatrelvir resistance in patients to-date, in vitro passaging of SARS-CoV-2 in the presence of increasing concentrations of nirmatrelvir yielded resistant viruses^63,64^. When drug resistant variants emerge in patients, new virus gene sequences and virus protein structures can be used to recompute the selection pressure of viral residues using the methods presented herein. In conclusion, we have presented a useful tool for antiviral development/screening by classifying the selection pressure of viral residues to evaluate if evolution of a given virus might diminish drug efficacy.

## Supplementary Information

Supplementary Table S1.

## Data Availability

The authors declare that the data supporting the findings of this study are available within the article and Supplementary Table S1 file.
