# Supplementary material for: A strategy for evaluating potential antiviral resistance to small molecule drugs and application to SARS-CoV-2
Source: Sci Rep. 2023 Jan 10;13:502. doi: 10.1038/s41598-023-27649-6 (PMC9831016; doi:10.1038/s41598-023-27649-6)
Supplement: Supplementary file 1 — Supplementary Table S1. [file 41598_2023_27649_MOESM1_ESM.pdf]

Supplementary Table 1. The list of SARS-CoV-2 inhibitor molecules, their viral protein targets, inhibitor-binding residues and the PDB IDs

Residues implicated drug binding	PDBs	Small molecule name	# of the compound
		**SARS-CoV-2, Mpro (3CLpro), nsp5**	
**C145, H41, E166, H164, T26, H163, F140, T25, C44, G143, N142;**	6LZE	11a	1
**C145, H164, E166, T26, H163, F140, *Q189*;**	6M0K	11b	2
**C145, H41, G143, M49, M165;**	7BUY	carmofur	3
**C145, H41, E166, G143, S144, H163, F140;**	6Y2F, 6Y2G	13b	4
**C145, H41, E166, H163, F140, H172, G143, *Q189,* H164, D187, T190, S144, N142, M49, M165;**	6WTT, 6WTJ, 7D1M, 7C6U, 7JSU, 7CBT	GC-376	5
**C145, H41, E166, G143, S144, M49, M165, H163, *R188*, *Q189*, F140**	6WTK	GC-373	6
**C145, H41, E166, G143, *Q192*;**	-	Indinavir	7
**C145, E166, F140, L141, H163, N142;**	7ADW	Tolperisone;	8
**C145, E166, F140, L141, H163, H164, N142;**	6YNQ	2-[β-(4-hydroxyphenyl)-ethylaminomethyl]-tetralone (HEAT)	9
**C145, H41, E166, G143, H163, R298;**	7AQJ	Triglycidyl isocyanurate	10
**C145, E166, *Q189*, H163, G143, H164;**	7LCS	1a	11
**C145, E166, H163, G143, H164;**	7LCO,	1i	12
**C145, E166, Q189, H163, G143, H164;**	7LDL	1f	13
**C145, E166, *Q189*, H163, G143, H164;**	7LCT	1g	14
**C145, H41, E166, G143, H164, *Q189*, M165, T190, Q192, M49, P168, L167, R188, D187, N142, L27, Y54;**	7BRP, 7C6S, 7COM	boceprevir	15

**C145, E166, G143, N142, *G302*, H164, *Q192*, *Q189*, H163;**	6LU7, 7BQY	N3	16
**C145, H41, E166, *Q189*, H164, H163, *D187*, *Q189*, F140, M165, H172, T190, A191, P168, G143;**	6XHM, 6XHL	PF-00835231	17
**H41, E166, F140, N142, L141, M165;**	-	manidipine	18
**C145, *Q189, Q192*, M165, L167, P168**	-	lercanidipine	19
**C145, H41, M165, P168, *A191*;**	-	efonidipine	20
**H41, N142;**	-	bedaquiline	21
**C145, H41, H163, H164, L141, F140, N142, E166, M165, T25, M49, C44, T24, S46, T45;**	7JU7, 7L5D	Masitinib	22
**C145, H41, E166, G143, H163, F140, T26, T45, C44, M49, N142, L141;**	7LTN	CDD-1713	23
**H41, E166, N119, T26, G143, T25, T24, C44, M165, S46, M49, H164, N142, Y237, *T199*, D289, *G195*, *A194*, L272 L287, Y239, L286, *T198*, *D197*, R131, K137, V171, T169;**	-	Glycyrrhizin	24
**C145, H41**	-	DNICs TGTA–RRE, [(μ-S-TGTA)Fe(NO)2]2 (TGTA = 1-thio-β-D-glucose tetraacetate) and TG–RRE, [(μ-S-TG)Fe(NO)2]2 (TG = 1-thio-β-D-glucose)	25, 26
**C145, H41, E166, H163, F140, L141, M165, S144, N142, G143, S144, M49, *D187*, R188, *Q189, T54*;**	-	Ethacrynic acid	27
**C145, H41, E166, H163, L141, H163, M165, G143, S144, N142, H164, M49, *D187, R188, Q189*;**	-	naproxen	28
**C145, E166, F140, L141, M165, S144, H164, N142, G143;**	-	allopurinol	29
**H41, E166, F140, L141, H163, M165, N142, G143, S144, H164, M49, *D187, R188, Q189*;**	-	butenafine hydrochloride	30
**C145, H41, F140, L141, H163, M165, L27, N142, G143, S144, H164, M49, *R188, Q189*, L167, P168;**	-	raloxifene	31
**H41, M165, H164, M49, *D187, R188, Q189*;**	-	tranylcypromine	32

**C145, H41, H163, E166, L141, M165, G143, S144, H164, N142, M49, *R188, Q189*;**	-	saquinavir mesylate	33
**C145, H41, M165, *R188, D187*, M49;**	-	Ebsulfur	34
**C145, E166, H163, G143, H164, H172, L167, P168, *Q192*, M165, M49;**	7JT7, 7JW8	compound 4	35
**C145, H41, M165, P39, H163, *D187*;**	7JT0	MAC-5576	36
**C145, H41;**	-	paritaprevir	37
**C145, *Q189*;**	-	tipranavir	38
**E166, N142;**	-	ombitasvir	39
**C145;**	-	ivermectin	40
**E166, N142;**	-	micafungin	41
**C145, N142, H163, M165;**	-	Vitamin K3	42
**C145, H41, N142, *Q189*, M165, P168, M49, H164, F294, P108, *I200*, V202, H246, T292, I249, P132, Q107, Q110;**	-	ebselen	43
**Q127, F3, K5, W207, E288;**	-	chebulagic acid (CHLA)	44
**D245, Q107, Q110**	-	punicalagin (PUG)	45
**E166, M165, S144, N142, M49, F140, L141, H164;**	-	Quercetin	46
**C145, H41, E166, G143, L141;**	-	Baicalein	47
**C145, H41, E166, G143, L141;**	-	wogonin	48
**E166, *Q192, Q189*;**	-	cilostazol	49
**C145, H41, E166, *Q189,* G143, N142, H163, H164, M165, L167, P168, *A191***	-	nelfinavir	50
**C145, H41, L141, S144, N142, G143, T26, L27, M165, F140;**	-	GRL-0920	51
**C145, E166, T26, S144, N142, L141, M165, H163, H172;**	-	GRL-0820	52
**C145, H41, *Q189*, T26, S144, G143, H164, *D187*, H163, L141;**	7DPP	myricetin	53
**C145, H41, E166, T25, T26, N119, M165, Y54, *T190, R188, Q189*;**	7DPU	Compound 3	54

**C145, H41, E166, T25, T26, N119, M165, Y54, *T190, R188, Q189*;**	7DPV	Compound 7	55
**C145, T26, N119;**	-	Re I Tricarbonyl Complexes	56
**C145, H41, E166, S46, C44, T25, L27, N142, *Q189*, M49;**	7L0D	ML188	57
**C145, H41, E166, *Q189*, H164, L27, M49, G143, P168, L27, H163**	7JKV	5h	58
**C145, H41, E166, F140, N142, H163, H172, M49, M165, *D187*, H164, *Q189,* P168;**	7JQ0	MPI3	59
**C145, H41, *Q189*;**	-	compound 2d	60
**C145, H41, E166, M49, M165, N142;**	-	compound 26	61
**C145, H41, E166, *Q189*, M49, N142, L141, H172, M165;**	-	Dalcetrapib	62
**C145, H41, E166, *R188, D187*, M49, H164, G143, H163, F140, S1, P168, L167, M65, *Q189*;**	7D3I	MI-23	63
**C145, H41, Q189, E166, G143, H164, N142, T26, D187, M49, Q192, R188, M165, T190, L167, P168;**	7C7P	Telaprevir	64
**C145, H41, E166, G143, N142, M165, P168, *Q189*, M49;**	6XBG	UAWJ246	65
**C145, H41, E166, *Q189*, M49, N142;**	6XBH	UAWJ247	66
**C145, H41, E166, G143, N142, P168, *Q189*, M49;**	6XBI	UAWJ248	67
**H41, V42, G143, N51, *V186*;**	-	niacin	68
**E166, V42, M165, L167;**	-	hit 1	69
**N277, T225;**	-	omeprazole	70
**C145, E166, *Q189,* H164, S144, H163, F140;**	7M2P	compound 18	71
**C145, H41, E166, H164, H163, F140, *Q189*, L167, P168, *A191, T190*;**	-	2a	72
**C145, H41, E166, H164, G143, *Q189*, H163, F140;**	-	2f	73
**C145, H41, E166, H164, H163, S144, F140, *Q189*;**	-	2k	74
**C145, H41, E166, H164, H163, S144, *Q189*;**	-	3d	75
**C145, H41, E166, H164, H163, *Q189*;**	-	3e	76
**C145, H41, E166,Q189, H164, H163, F140;**	6XMK	7j	77

**H41, E166, *Q189,* H164, M49, H164, *A191*, C44, T26, T25, *Q192*, P168, F185, V186;**	-	Bisindolylmaleimide IX	78
**K12, K97, K100, Y101, F103, K102, V104, R105;**	-	Suramin	79
**C145, H41, *D187,* M49, M165, *A194,* V186, *Q189, R188, D187*;**	-	Quinacrine	79
**C145, H41, E166, N142, G143, H164, M165, S144, *Q192*, P168;**	7D1O	narlaprevir	80
**C145, H41, E166, P168, H163, F140, H164, M49, M165, *Q189, Q192;***	-	azapeptide nitrile 8	81
**C145, H41, E166, Q189, D187, R188, G143, M49, Y54, M165, H163, Q192, F140, S144, N142;**	7VH8	PF-07321332	82
**H41, E166, C44, Q192, Q189,**	-	M3	83
**C145, H41, E166, H163, Q189, Q192, M49, R188, M165, T26, L141, F140, T25;**	7L14, 7L13, 7L12, 7L11, 7M8P	Compound 5	84
**C145, E166, G143, H163, T26, H164, M165, R188, Q189, T190, P168;**	7L14, 7L13, 7L12, 7L11, 7M8P	Compound 14 (2603242-04-0)	85
**C145, H41, E166, G143, T26, H163, M165, T190, P168, Q189, M49, T25;**	7L14, 7L13, 7L12, 7L11, 7M8P	Compound 21 (2603242-35-7)	86
**C145, H41, E166, G143, H163, T26, H164, Q189, T190;**	7L14, 7L13, 7L12, 7L11, 7M8P	Compound 23 (2603242-41-5)	87
**C145, H41, E166, H163, M165, T190, Q189, H164, M49, T25, T26, N142, L141;**	7L14, 7L13, 7L12, 7L11, 7M8P	Compound 26	88
**C145, E166, H163, C44, T25, S46, M49, N142, L141, S1, F140, M165, H164;**	7LMF	compound 2694063-46-0 (21)	89
**H41, E166, G143;**		81418-42-0 (15)	90
**H41, E166, N142, M49, F140, L141;**		392732-12-6 (13)	91
**T24, T25, Q189, N142, M49, L141**		compound 7	92

**C145, H41, E166, H163, M165;**		PET-UNK-29afea89-2	93
**C145, H41, E166, H163, N142, S144, G143, M165, R188, T190, A191, Q189**	7MBI	15l	94
		**SARS-CoV-2 Plpro**	
**Q269, Y268, P248, Y264, L162, P247, T301, Y273;**	-	rac5c	95
**E167, Q269, Y268, D164, Y273, Y264, P247, P248; T301, D164**	7JIR, 7CMD	1/GRL-0617	96
**K157, E167, Q269, Y268, D164, Y264, P247;**	7JIT	2	97
**K157, E167, D164, Y264, P247, Y268, Q269;**	7JIV	3	98
**Q269, D164;**	7JIW	4	99
**P248, Y264, Y273, Q195, T225, C192, C226, F69, H73, Y268;**	7D7L	YM155	100
**M208, L162, G163, E161, Y268, D164, N267, Y273, Y264, Y268, T301, P247, P248**	7E35)	12, 19	101, 102
**D164, E167, Q269, Y268, P248, N267**	7LLF, 7LLZ, 7LOS	XR8-65, XR8-69, XR8-83	103, 104, 105
**D164, E167, Q269, Y268, P248, N267, P299**	7LBS	XR8-24	106
**T301, D164, Y273, G163, C111, N109, L162, H272, G271, Y268, Y264, P248, P247;**	6WUU	VIR250	107
**D164, T301, G163, C111, W106, N109, L162, G271, Y264, Q269, P248**	6WX4	VIR251	108
**C189, C192**		ebselen	43
**C189, C192**		disulfiram	109
**K157, E167, P248, Y264**		392732-12-6 (13)	91
**K157, P248, Y264, Y268**		compound 7	92
		**SARS-CoV-2 spike protein**	
**Y453, R403, D405, R408**	-	glycyrrhizic acid (ZZY-44)	110
**L849, A829, P1213, Y1209, V826, I1210, K1205, L1203**	-	Eltrombopag	111

**R403, Y449, Y453, Q493, S494, Q498, G502, Y505, Y495, G496, T500, N501;**	-	Geraniin	112
**S494, Y505;**	-	ceftazidime	113
**C336, F338, G339, F342, F374, S373, S371, L366, D364;**	-	ursolic acid (1)	114
**S371, L366, A363, V362, F338, G339;**	-	quercetin (7)	46
**F374, N343, G339, F338;**	-	1, 2, 3, 4, 6-penta-O-galloyl-β-d-glucose (12).	115
**K458, R457, R466, R355, R357, K356, K444, R346, N343;**	-	Inorganic polyphosphate (polyP)	116
**T415, G416, K417, R403, D405, K417;**	-	compounds (4) and (5)	117, 118
**G504, Y505, Q498, Y495, Q493, S494, G496, Y449;**	-	Corilagin	119
**R454, L492, P491, L455, K458, E471, F456, R457, T470;**	-	1,3,6-Tri- O-galloy-β-D-glucose (TGG)	120
**Y505, D405, D405, E406, R403;**	-	MU-UNMC-1 and MU-UNMC-2	121, 122
**F377, C379, Y380, K378, R408, T376, P384;**	-	Betulinc acid, glycyrrethinic acid, oleanolic acid and potassium canrenonate, BAR704, BAR501, and BAR502	122-128
		**RNA-dependent RNA polymerase, nsp-12**	
**C813, L758, D761, D760, R555, S682;**	-	lycorine, b emetine, c cephaeline	129, 130, 131
**M542, A558, T556, D623, R553, R555**	-	I-13e	132
**N496, N497, K500, R569, Q573, K577, G590, L576, A580, A685, Y689, L758, A550, K551, R555, R836, R553, D856, H439, I548, S549, A840, S861, L862;**	7D4F	suramin	79
**R555, V166;**	-	ledipasvir	133
**K545, R555, D760, D623, S682, T687, S759, N691;**	7BV2	remdesivir	134
**R555, D623, S682, T687, D760;**	-	GS441524	135

**K545, N691, S682, R553, R555, K798, K621, D623, D761;**	7AAP, 7CTT	Favipiravir	136
**L460, P461, F321, V320, T268, S255, I266, P322, P323, R349, P677, N628**	-	Taroxaz-104	137
**D623, N691, S759;**	-	Lycorine	138
**R624, Y455, R553, K621, D623, Y619, C622, N691, S682, S795, P620, D618, V166;**	-	CoViTris2020	139
**D418, N416, F415, K849, D846, K411, S15, K417, N691, D623, P412;**	-	ChloViD2020	140
**E Protein**			
**I46, L51, P54, S60, C44, V47, L31, L34, C40**	-	ZINC23221929	141
**S60, V47, C40, L28, L31, Y57**	-	ZINC06220062	142
**N15, T11, L12, E8**	-	proanthocyanidins	143
**Helicase nsp13**			
**A313, A316, E375, K320, S289, G287, Q537**	-	Cepharanthin	144
	-	ebselen/disulfiram	43, 109
**uridylate-specific endoribonuclease (EndoU) enzyme nsp-15**			
**T341, G248, Q245, K290, H250, C293, S294, L346P344, Y343, T341, H235**	-	Tipiracil	145
**nucleoprotein (N protein)**			
**W52, R149, I146, T76, N77, S79 and H145, I157, L104, Y112, R92, R107, R93 and Y109, Y111, residues stabilizing the association of RNA with N NTD or AMP**	-	Naproxen	146
**exoribonuclease/methyltransferase (nsp14) and its cofactor (nsp10)**			
**W385, F401, Y420, F426, F506, N386, R310, G333, Ile338, K336, H424, P335, D382**	-	(compound 13) bearing a 4-chloro-3-nitrobenzenesulfonamide moiety in the N-linker between both adenosines	147

**C189, C192**	-	ebselen/disulfiram	43, 109
**Nsp3 macrodomain CoV-2 Mac1**			
**I23, N40**	-	PARG-345	148
**2′-O-RNA methyltransferase (MTase) Nsp-16**			
**F6947, D6912, L6898, C6913, M6928, G6871, D6897, N6899, D6928, Y6845, N6841**	6WKQ	sinefungin (SFG)	149
